# Paracrine-Mediated Neuroprotection and Neuritogenesis of Axotomised Retinal Ganglion Cells by Human Dental Pulp Stem Cells: Comparison with Human Bone Marrow and Adipose-Derived Mesenchymal Stem Cells

**DOI:** 10.1371/journal.pone.0109305

**Published:** 2014-10-07

**Authors:** Ben Mead, Ann Logan, Martin Berry, Wendy Leadbeater, Ben A. Scheven

**Affiliations:** 1 Neurotrauma Research Group, Neurobiology Section, School of Clinical and Experimental Medicine, University of Birmingham, Birmingham, United Kingdom; 2 School of Dentistry, University of Birmingham, Birmingham, United Kingdom; National Institutes of Health, United States of America

## Abstract

We have investigated and compared the neurotrophic activity of human dental pulp stem cells (hDPSC), human bone marrow-derived mesenchymal stem cells (hBMSC) and human adipose-derived stem cells (hAMSC) on axotomised adult rat retinal ganglion cells (RGC) *in vitro* in order to evaluate their therapeutic potential for neurodegenerative conditions of RGC. Using the transwell system, RGC survival and length/number of neurites were quantified in coculture with stem cells in the presence or absence of specific Fc-receptor inhibitors to determine the role of NGF, BDNF, NT-3, VEGF, GDNF, PDGF-AA and PDGF-AB/BB in stem cell-mediated RGC neuroprotection and neuritogenesis. Conditioned media, collected from cultured hDPSC/hBMSC/hAMSC, were assayed for the secreted growth factors detailed above using ELISA. PCR array determined the hDPSC, hBMSC and hAMSC expression of genes encoding 84 growth factors and receptors. The results demonstrated that hDPSC promoted significantly more neuroprotection and neuritogenesis of axotomised RGC than either hBMSC or hAMSC, an effect that was neutralized after the addition of specific Fc-receptor inhibitors. hDPSC secreted greater levels of various growth factors including NGF, BDNF and VEGF compared with hBMSC/hAMSC. The PCR array confirmed these findings and identified VGF as a novel potentially therapeutic hDPSC-derived neurotrophic factor (NTF) with significant RGC neuroprotective properties after coculture with axotomised RGC. In conclusion, hDPSC promoted significant multi-factorial paracrine-mediated RGC survival and neurite outgrowth and may be considered a potent and advantageous cell therapy for retinal nerve repair.

## Introduction

The axons of retinal ganglion cells (RGC) transmit action potentials along the optic nerve to the superior colliculus (SC) and lateral geniculate nucleus (LGN) that are relayed onwards to the visual cortex. Axotomised RGC die [1], [2] so that blindness ensues after traumatic (crush or transection) [3] optic nerve injury. RGC loss is caused by a failure in the supply of neurotrophic factors (NTF; including neurotrophins), retrogradely transported from the SC/LGN neurons, that act as survival signals, ensuring the functional integrity of RGC connections [4]–[6].

As well as protecting RGC from death, NTF have the potential to promote the regeneration of transected axons and establish re-connection with their targets. The paucity of NTF in the central nervous system (CNS) is one explanation for the lack of axon regeneration compared to the peripheral nervous system (PNS) [2], [7] in which successful and functional axon regeneration occurs, largely promoted by Schwann cell-derived NTF [8]. Attempts to promote long distance axon regeneration by the transplantation of peripheral nerve grafts into the CNS have met with some success [9]. For example, grafting a peripheral nerve into the vitreous body after optic nerve crush [8] promotes more RGC axon regeneration in the transected optic nerve than occurs after the removal of Schwann cells before transplantation, suggesting that axotomised RGC regenerate their axons when provided with a constant supply of NTF. However, single NTF supplementation [7], or single dose treatments of NTF such as BDNF [10], [11], have proven unsuccessful and sustained delivery of multiple NTF to RGC over extended periods of time is difficult to achieve.

The vitreous is a relatively accessible immunoprivileged transplantation site [12] that lies directly adjacent to the RGC layer of the retina, allowing diffusion or transport of NTF across the inner limiting membrane to the RGC. Previously, we used intravitreally transplanted genetically modified fibroblasts expressing FGF-2, BDNF and NT-3 to promote RGC survival and axon regeneration after optic nerve crush [7]. Since the translational potential of genetically modified cells is limited, mesenchymal stem cells (MSC), which secrete a large array of NTF, have gained credence as a potential cell therapy for diseased and injured CNS neurons. Human bone marrow-derived mesenchymal stem cells (hBMSC) protect RGC from death in both optic nerve crush [13] and glaucoma experimental models [14]–[16] through the production of NTF (e.g. platelet-derived growth factor (PDGF) [15]), without differentiation of hBMSC into replacement RGC/RGC-like cells.

We recently demonstrated that rat dental pulp stem cells (DPSC) protected adult rat RGC from death in an optic nerve crush model [17], [18]. This effect was significantly greater than that achieved by rat BMSC and mediated through nerve growth factor (NGF), brain-derived neurotrophic factor (BDNF) and neurotrophin 3 (NT-3) via, TrKA, B and C receptor signalling. Our findings were consistent with previous studies showing significant expression [19], [20] and secretion of NGF, BDNF and NT-3 by hDPSC [21]. The neuroprotective and axogenic properties of DPSC [17], [18] and BMSC [14], [15], [22] can also be found in other MSC types, in particular adipose-derived mesenchymal stem cells (AMSC) that also secrete multiple NTF [22], [23] and promote functional recovery after CNS trauma including spinal cord injury [22], [24], stroke [25] and light induced photoreceptor damage [26], [27]. However, AMSC have not been tested in a model of RGC death. Comparative analyses of different human MSC is still lacking although important for the determination of the most efficacious paracrine-mediated therapy for the injured retina. Thus, the aim of this study was to evaluate and compare the neuroprotective and neuritogenic effects of hDPSC, hBMSC and hAMSC and to define the stem cell NTF secretome using ELISA and PCR microarray analysis.

## Materials and Methods

All reagents were purchased from Sigma (Poole, UK) unless otherwise specified.

### DPSC/BMSC/AMSC cultures

hDPSC were purchased from AllCell LLC (Berkeley, CA) and both hBMSC and hAMSC from Lonza (Slough, UK), and each represented pooled samples from 3 donors. The CD29^+^/CD44^+^/CD73^+^/CD90^+^/CD45^−^ (confirmed by supplier) stem cells were seeded into T25/T75 flasks (Corning, Amsterdam, NL) in both a total volume of 5 ml/15 ml DMEM containing 1% penicillin/streptomycin and 10% foetal bovine serum (FBS; Hyclone Laboratories, Logan, UT) and at a density of 1×10^6^ cells/2×10^6^ cells, respectively. Cultures were maintained at 37°C in 5% CO_2_, the supplemented medium was changed every 3 d and the cells were passaged when 80% confluent using 0.05% trypsin/EDTA to lift them from their surface attachment.

### Animals

Nine adult male Sprague-Dawley rats weighing 170–200 g (Charles River, Kent, UK) were housed under Home Office guidelines in conditions of 21°C and 55% humidity under a 12 h light and dark cycle, given food/water *ad libitum* and were under constant supervision from trained staff. Animals were killed by “Schedule 1 Methods” i.e. rising concentrations of CO_2_ before extraction of retinae. Ethical approval by the University of Birmingham ethical review Sub-Committee for this study was not required due to the *in vitro* nature of the experiment.

### Retinal cell coculture

Cell culture 24-well plates (BD Biosciences, Oxford, UK) were pre-coated with 100 µg/ml poly-D-lysine for 60 min and then with 20 µg/ml laminin for 30 min. After culling and ocular dissection, the retinae of nine male Sprague-Dawley were minced in 1.25 ml of papain (20 U/ml; Worthington Biochem, Lakewood, NJ; as per manufacturer’s instructions) containing 50 µg/ml of DNase I (62.5 µl; Worthington Biochem) and incubated for 90 min at 37°C. The retinal cell suspension was centrifuged at 300×g for 5 min and the pellet resuspended in 1.575 ml of Earle’s balanced salt solution (Worthington Biochem) containing 1.1 mg/ml of reconstituted albumin ovomucoid inhibitor (150 µl; Worthington Biochem) and 56 µg/ml of DNase I (75 µl). After adding to the top of 2.5 ml of albumin ovomucoid inhibitor (10 mg/ml) to form a discontinuous density gradient, the retinal cell suspension was centrifuged at 70×g for 6 min and the cell pellet resuspended in 1 ml of supplemented Neurobasal-A (25 ml Neurobasal-A (Life Technologies, Gibco, Paisley, UK), 1X concentration of B27 supplement (Life Technologies, Invitrogen, Paisley, UK), 0.5 mM of L-glutamine (62.5 µl; Invitrogen) and 50 µg/ml of gentamycin (125 µl; Invitrogen)) and seeded at a density of 125,000 cells/800 µl/well in a 24 well plate. Previous immunocytochemical analysis of these cultures in our lab demonstrates that 60% of these retinal cells are neurons (neurofilament^+^/βIII-tubulin^+^), of which 10% are Thy1^+^ RGC [28].

hDPSC, hBMSC and hAMSC were used at passage 2–4 and plated at a density of 50,000 cells/200 µl onto a 0.4 µm porous cell culture insert (Millipore, Watford, UK) that was placed into each of the 24 wells containing retinal cells to give a total volume of 1 ml of medium/well. Particular wells containing retinal cell cultures were treated either singly or in combination with 5 µg/ml Fc-TrKA, Fc-TrKB and/or Fc-TrKC (R&D Systems, Abingdon, UK) as well as Fc-VEGFr, Fc-GDNFr, Fc-PDGFAr and Fc-PDGFBr fusion protein inhibitors [29] (R&D Systems) which are highly specific inhibitors for the corresponding cognate neurotrophic factor receptors (NTFR). A combination of recombinant human NGF, BDNF and NT-3 was also added to some retinal cell cultures at 60 ng/ml as a positive control. Particular wells containing retinal cells were treated with 0.1 µm, 1 µm and 10 µm of VGF (R&D Systems) instead of hDPSC/hBMSC/hAMSC.

Cocultures were incubated for 3 d at 37°C before immunocytochemical staining of RGC with βIII-tubulin. For this study, large spherical βIII-tubulin^+^ retinal cells [30] cultured after neuronal isolation from retinae, are referred to as RGC. βIII-tubulin is a reliable and relatively specific marker for RGC [30], although cross reactivity with amacrine cells has been suggested [31]. However, it should be emphasised, that the isolation procedure and growth medium used preferentially selects and yields populations of neuronal cells, we are confident that our findings accurately reflect RGC numbers. All experiments were repeated on 3 separate occasions. Each of the treatment groups in each of the 3 experimental runs comprised 3 replicate wells containing retinal cells harvested from one animal.

### Immunocytochemistry

Retinal cells in 24 well plates (BD Biosciences) were fixed in 4% paraformaldehyde (PFA) in phosphate-buffered saline (PBS) for 10 min, washed for 3×10 min of PBS, blocked in blocking solution (0.5% bovine serum albumin (g/ml), 0.3% Tween-20, 15% normal goat serum (Vector Laboratories, Peterborough, UK) in PBS) for 20 min and incubated with primary antibody (anti-rat βIII-tubulin, raised in mouse, #T8660 diluted at 1∶500 in antibody diluting buffer (ADB; 0.5% bovine serum albumin, 0.3% Tween-20 in PBS) for 1 h at room temperature. Cells were then washed for 3×10 min in PBS, incubated with the secondary antibody (anti-mouse IgG Fluor 488, raised in goat, 1∶400, #A-11001; Life Technologies, Molecular Probes, Paisley, UK) diluted in ADB for 1 h at room temperature, washed for 3×10 min in PBS, mounted in Vectorshield mounting medium containing DAPI (Vector Laboratories) and stored at 4°C.

### Microscopy and analysis

For immunocytochemistry, all βIII-tubulin^+^ RGC, with or without neurites, were counted in each well of the 24 well plates, recording the total number of RGC and the number of RGC with neurites. Neurite outgrowth was measured in images taken at 20X magnification using an Axiocam HRc camera (Carl Zeiss Ltd, Hertfordshire, UK). Each well was divided into 9 equal sectors and the length of the longest neurite of each RGC in each sector was measured using Axiovision software (Carl Zeiss Ltd). Fluorescently stained cells were analysed by an operator blinded to treatment groups, using a Zeiss Axiovert fluorescent microscope (Carl Zeiss Ltd).

### NTF ELISA

To quantify the growth factors and NTF produced by hDPSC, hBMSC and hAMSC, conditioned medium was collected from cultures at passage 2 and 5 after 48 h in culture and assayed using a duoset ELISA kit for human NGF, BDNF and NT-3, VEGF, GDNF, platelet-derived growth factor (PDGF-AA) and PDGF-AB/BB according to the manufacturer’s instructions (R&D Systems). Briefly, a standard curve was constructed using NTF standards and test samples of conditioned medium at increasing dilutions, run in duplicate with NTF concentrations extrapolated from the standard curve. Values were normalized to the number of cells in the flask (manually counted *via* haemocytometer) and the volume of medium, and corrected for analyte found in the medium/serum.

### Human NTF and NTFR PCR array

The expression of 84 NTF and NTFR genes by hDPSC, hBMSC and hAMSC (passage 2) was assayed using quantitative RT-PCR profiler arrays (PAMM-031) by a commercially run service (Sabiosciences, Qiagen, Hilden, Germany). Housekeeping genes (*b2m* and *hprt1*) were used to normalize the data and the ^–ΔCt^ compared between cell types and expressed as + or - fold changes. Samples contained one million cells and were run in triplicate.

### Statistics

All statistical tests were performed using SPSS 17.0 (IBM SPSS, Inc., Chicago, IL) and data presented as mean ± standard error of the mean (SEM). The Shapiro-Wilkes test was used to ensure all data were normally distributed before parametric testing using a one-way analysis of variance (ANOVA) with a Tukey *post-hoc* test. Statistical differences were considered significant at p values <0.05. For the qRT-PCR, data were compared by a Student’s t test and statistical significance set at p<0.001.

## Results

### hDPSC promoted significantly greater paracrine-mediated neuroprotection and neuritogenesis than hBMSC/hAMSC

**Figure 1:**
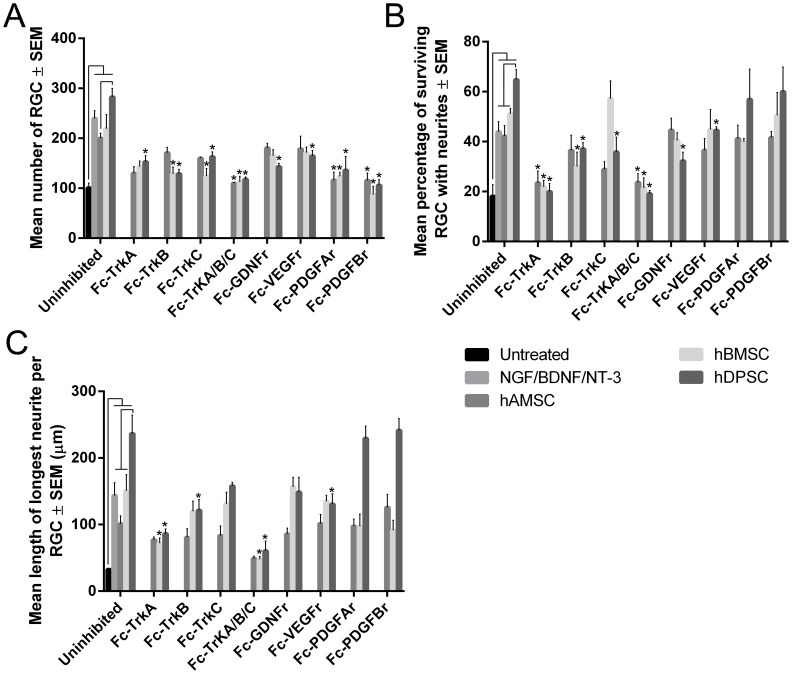
Effects of hDPSC, hBMSC and hAMSC on axotomised RGC in culture. The number of RGC (**A**), percentage of surviving RGC bearing neurites (**B**) and the length of the longest RGC neurite (**C**) in both untreated retinal cultures and after coculture with hDPSC, hBMSC, hAMSC, with or without added exogenous neurotrophins. *Black lines* indicate significant difference at *P*<0.05. The effects of Fc-TrKA, -B, -C, Fc-GDNFr, Fc-VEGFr, Fc-PDGFAr and Fc-PDGFBr inhibitors on RGC survival (**A**) and neuritogenesis (**B, C**) in hDPSC, hBMSC and hAMSC cocultures are also shown (values marked with an *asterisk* indicate significant difference from uninhibited cultures at *P*<0.05).

**Figure 2:**
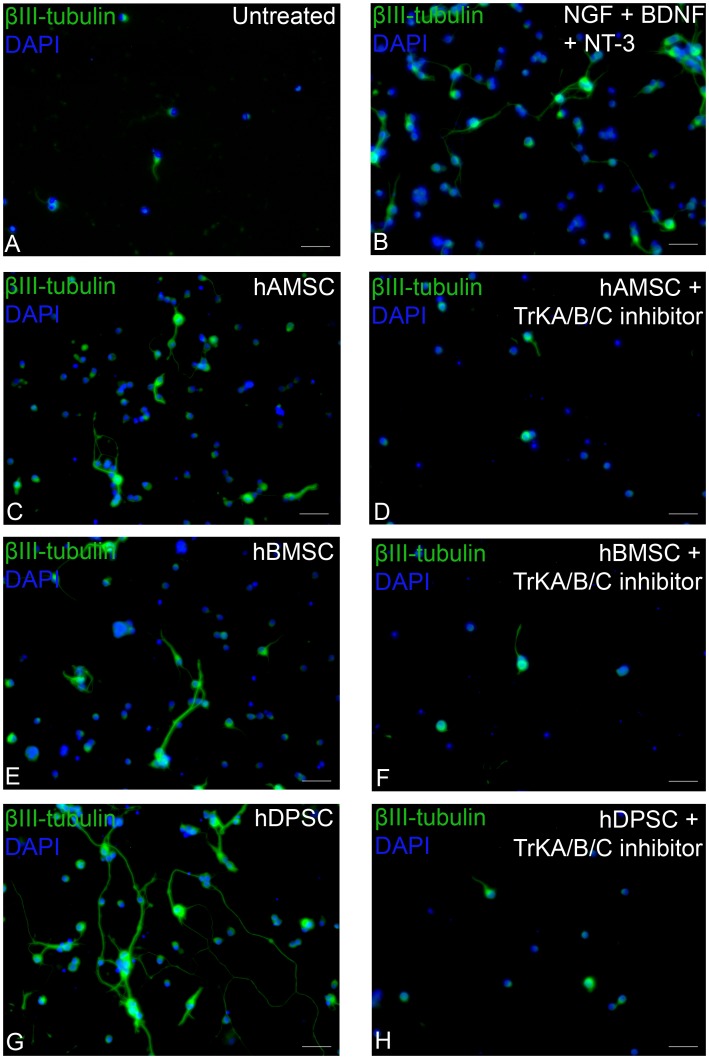
Immunocytochemical staining of RGC after retinal coculture with hDPSC, hBMSC and hAMSC in transwell inserts. *In vitro* RGC cultured either alone (**A**), with exogenous neurotrophins (**B**), with hAMSC (with or without TrK inhibitors (**C**, **D**, respectively)), with hBMSC (with or without TrK inhibitors (**E**, **F**, respectively)) or with hDPSC (with or without TrK inhibitors (**G**, **H**, respectively). All images are representative of the entire culture, nine separate culture wells/treatment, with every three wells using a different animal (*scale bars*: 50 µm), cell nuclei were stained with DAPI.

hDPSC, hBMSC and hAMSC all promoted a significant increase (p<0.05) in the survival of cocultured RGC (282.7±17.1 cells/well; 219±28.4 cells/well; 200.0±10.2 cells/well; respectively) compared with retinal cells cultured alone (100.7±9.5 cells/well); hDPSC, hBMSC and hAMSC neuroprotection was similar to the level obtained after retinal cell culture with recombinant NGF/BDNF/NT-3 (239.7±15.4 cells/well; Figs. 1, 2.). The increase in survival of RGC in hDPSC-treated retinal cultures was significantly greater (p<0.05) than that achieved in cocultures with hAMSC (p<0.05) but not significantly greater than that seen in cocultures with hBMSC.

Coculture of retinal cells with hDPSC, hBMSC and hAMSC significantly increased (p<0.05) the percentage of surviving RGC bearing tubulin^+^ neurites (64.8±4.0%, 51.1±2.1%, 42.2±4.3%, respectively), as well as the length of neurites (236.7 µm±27.6 µm, 150.7 µm±24.1 µm, 101.1 µm±12.1 µm, respectively) compared with retinal cells cultured alone (18.2±4.6%; 32.8 µm±1.4 µm; Fig. 1, 2.). Coculture with hDPSC promoted a significant increase (p<0.05) in the number of neurite-bearing RGC as well as neurite length when compared with cocultures with hBMSC and hAMSC, or with retinal cultures exposed to recombinant NGF, BDNF and NT-3. These data confirm that hDPSC, hBMSC and hAMSC have paracrine-mediated neuroprotective and neuritogenic properties, with hDPSC promoting the most significant effects.

### NTFR Fc-receptor blockers for multiple NTFR attenuated the neuroprotective and neuritogenic effect of hDPSC/hBMSC/hAMSC

The NTFR blockers Fc-TrKA, Fc-TrKB, Fc-TrKC, Fc-TrKA/B/C, Fc-GDNFr, Fc-VEGFr, Fc-PDGFAr and Fc-PDGFBr significantly attenuated hDPSC mediated neuroprotection and/or neuritogenesis of cocultured RGC (Table. 1; Fig. 1, 2.) compared to uninhibited hDPSC/retinal cell cocultures. Fc-TrKA, Fc-TrKB, Fc-TrKC, Fc-TrKA/B/C, Fc-PDGFAr and Fc-PDGFBr significantly attenuated hBMSC mediated neuroprotection and/or neuritogenesis of cocultured RGC compared to uninhibited hBMSC/retinal cell cocultures. Fc-TrKA/B/C, Fc-PDGFAr and Fc-PDGFBr significantly attenuated hAMSC mediated neuroprotection and/or neuritogenesis of cocultured RGC compared to uninhibited hAMSC/retinal cell cocultures. These data demonstrate that the neuroprotective and neuritogenic effects afforded by each of the stem cell types are mediated through a variety of different NTF.

**Table 1:** The number of surviving RGC (cells/well), percentage of surviving RGC growing neurites (%) and the length of the longest neurite (µm) after coculture of RGC with hDPSC, hBMSC or hAMSC and inhibition with Fc-TrKA, Fc-TrKB, Fc-TrKC, Fc-TrKA/B/C, Fc-GDNFr, Fc-VEGFr, Fc-PDGFAr and Fc-PDGFBr.

Treatment		Without inhibition	Fc-TrKA	Fc-TrKB	Fc-TrKC	Fc-TrKA/B/C	Fc-GDNFr	Fc-VEGFr	Fc-PDGFAr	Fc-PDGFBr
hDPSC	RGC/well	282.7±17.1	**153.0±12.1**	**129.3±8.5**	**163.3±9.4**	**118.3±4.3**	**143.3±6.6**	**164.7±10.7**	**136.3±27.1**	**106.0±11.3**
	% surviving RGC with neurites	64.8±4.0	**20.0±3.2**	**37.1±2.4**	**35.9±5.8**	**19.2±1.3**	**32.3±3.4**	**44.5±1.4**	57.0±12.1	60.1±9.7
	Length of longest neurite (µm)	236.7±27.6	**86.4±7.0**	**121.6±16.1**	158.2±5.1	**60.6±14.8**	148.9±22.2	**130.8±15.4**	229.5±18.4	241.6±17.6
hBMSC	RGC/well	219±28.4	143.0±11.4	**128.7±13.6**	**124.0±15.4**	**112.7±11.0**	165.3±11.9	171.0±11.6	**123.7±8.0**	**87.7±16.1**
	% surviving RGC with neurites	51.1±2.1	**21.9±2.5**	**30.1±5.7**	57.3±7.0	**21.4±4.1**	40.5±3.0	44.83±8.0	39.9±1.4	50.6±9.0
	Length of longest neurite (µm)	150.7±24.1	**72.0±7.8**	119.9±15.3	131.1±17.6	**47.9±4.2**	156.7±14.2	135.0±8.9	97.4±18.2	91.3±15.0
hAMSC	RGC/well	200.0±10.2	130.0±13.1	171.3±10.4	160.0±2.6	**110.3±1.2**	165.3±11.9	171.0±11.6	**116.3±15.9**	**115.7±14.5**
	% surviving RGC with neurites	42.2±4.3	**23.5±4.8**	36.6±6.0	29.0±3.0	**23.7±3.5**	44.6±4.7	36.6±4.7	41.3±5.3	41.6±2.4
	Length of longest neurite (µm)	101.1±12.1	77.1±4.3	80.8±13.1	83.6±14.2	49.1±3.6	156.7±14.2	135.0±8.9	97.6±10.5	125±19.5

Values significantly (p<0.05) different from coculture without inhibition are given in bold (Mean +/− SEM; n = 3).

### hDPSC, hBMSC and hAMSC have distinct NTF expression profiles

**Figure 3:**
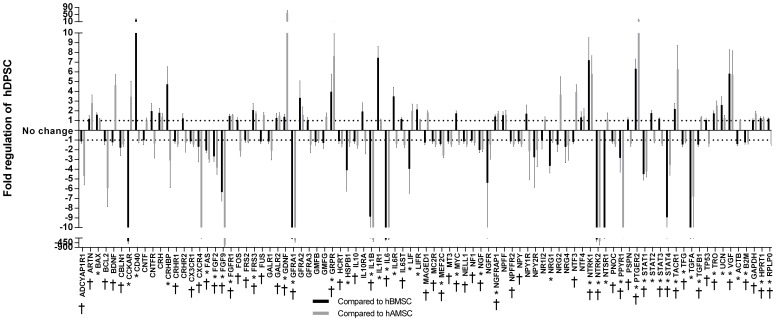
Expression of NTF and NTFR genes by hDPSC, hBMSC and hAMSC. RT-PCR array analysis of 84 genes encoding NTF and NTFR expressed by hDPSC, displayed as fold regulation in comparison to either hBMSC (black bars) or hAMSC (grey bars). The horizontal dotted lines represent fold-changes of ±1 (no difference). Significant differences between hDPSC and hBMSC (*) and hDPSC and hAMSC (†) at p<0.05 are labelled on the x axis.

The PCR array detected 84 NTF and NTFR genes differentially expressed by hDPSC, hBMSC and hAMSC. For example, hDPSC express ≥4 fold higher *cd40, crhbp, grpr, il1r1, ntrk1, ptger2* and *vgf* than hBMSC and ≥4 fold higher *bdnf, gdnf, grpr, nt-3, ptger2, tacr1* and *vgf* than hAMSC (Fig. 3.). hDPSC express ≥4 fold lower *cckar, fgf9, gfra1, hspb1, il1b, il6, ngfr, ntrk2, ntsr1, stat1, stat4 and tgfa* than hBMSC and ≥4 fold lower *adcyap1r1, bcl2, cxcr4, fgf9, gfra1, il1b, il6, ntrk2, ppyr1, stat1, tgfa* than hAMSC. Significant differences (p<0.001) are highlighted in the graph (Fig. 3). These data confirm that, despite hDPSC, hBMSC and hAMSC all being designated as mesenchymal stem cells, the NTF secretome is distinct between each stem cell phenotype.

### hDPSC secrete multiple NTF at higher levels than hBMSC/hAMSC

**Figure 4:**
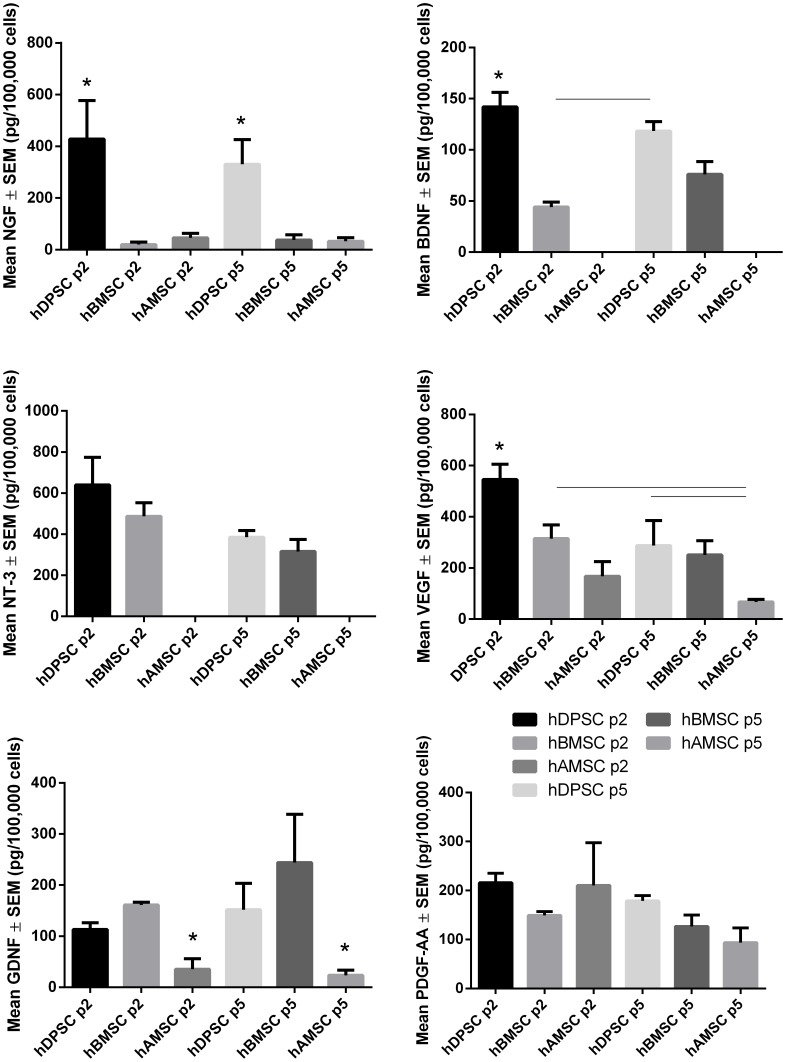
NGF, BDNF, NT-3, CNTF, VEGF, GDNF, PDGF-AA and PDGF-AB/BB titres by ELISA in hDPSC, hBMSC and hAMSC. hDPSC-, hBMSC- and hAMSC-conditioned media (passage 2 and 5) collected after 48 h of cell culture. (n = 3; *asterisks* indicate significant differences from all other samples/*black lines* indicate significant difference between specific samples at p<0.05). CNTF and PDGF-AB/BB were undetectable in all samples.

The levels of secretion by hDPSC, hBMSC and hAMSC of NGF, BDNF, NT-3, VEGF, GDNF, PDGF-AA, PDGF-AB/BB and CNTF, at passage 2 and 5, are detailed in Table 2, and presented as pg/100,000 cells/48 h. CNTF and PDGF-AB/BB were undetectable in hDPSC/hBMSC/hAMSC conditioned media, while BDNF and NT-3 were undetectable in hAMSC conditioned medium. The hDPSC secreted significantly greater (p<0.05) titres of NGF, BDNF and VEGF than hBMSC/hAMSC (Table. 2; Fig. 4.). These data confirm that the hBMSC, hAMSC, and in particular, hDPSC secrete a variety of different NTF.

**Table 2:** The titre of secreted NGF, BDNF, NT-3, VEGF, GDNF, PDGF-AA, PDGF-AB/BB and CNTF in conditioned media from hDPSC, hBMSC and hAMSC at passage 2 and 5, as determined by ELISA.

		NGF	BDNF	NT-3	VEGF	GDNF	PDGF-AA	PDGF-AB/BB	CNTF
hDPSC	Passage 2	428.0±149.0	141.9±14.4	639.8±134.9	314.7±53.9	113.2±13.1	216.0±19.0	0	0
	Passage 5	331.2±94.8	118.3±9.2	385.3±32.6	287.3±98.4	151.7±51.6	178.9±11.0	0	0
hBMSC	Passage 2	20.4±9.5	44.1±4.8	487.4±65.4	314.7±53.9	161.2±5.2	149.2±7.9	0	0
	Passage 5	37.7±20.7	76.0±12.6	316.2±58.3	251.2±54.9	244.3±94.2	126.8±23.2	0	0
hAMSC	Passage 2	46.1±17.5	0	0	167.6±57.2	35.5±20.2	210.3±87.1	0	0
	Passage 5	33.2±14.0	0	0	66.7±10.5	23.6±10.0	93.9±29.8	0	0

Data are presented as pg/100,000 cells/48 h (Mean +/− SEM; n = 3).

### VGF was neuroprotective for RGC

**Figure 5:**
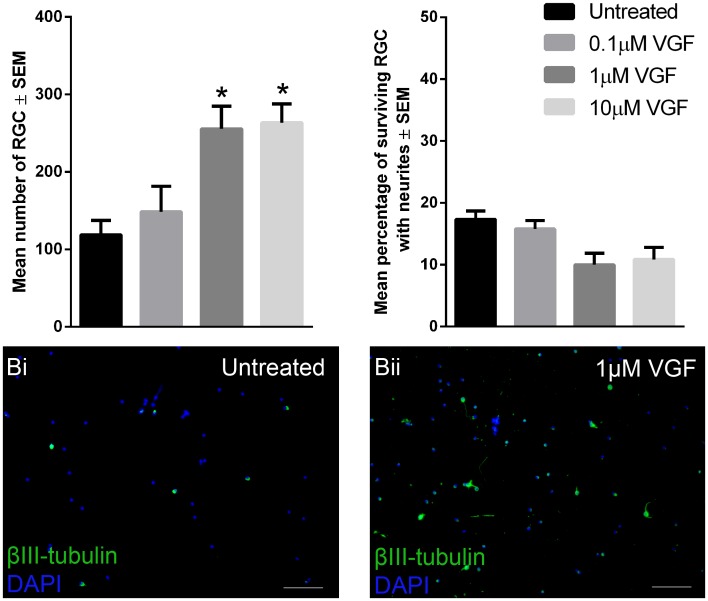
The effects of VGF on RGC in retinal cultures. The total number of surviving RGC as well as the percentage of RGC bearing neurites either cultured alone, or with 0.1 µM, 1 µM and 10 µM VGF (**A**). *Asterisks* indicate significant difference from other treatment groups at p<0.05. RGC cultured either alone (**Bi**) or with 1 µm VGF (**Bii**). All images are representative of the entire culture, nine separate culture wells/treatment with every three wells using different animals (*scale bars*: 100 µm), cell nuclei were stained with DAPI.

The differentially and significantly greater transcription of *vgf* in hDPSC compared to hBMSC/hAMSC (Fig. 3.) led to investigation of the neuroprotective and/or proregenerative properties of VGF. VGF promoted a significant increase (p<0.05) in the survival of cultured RGC at concentrations of 1 µM (255.5±29.4 cells/well) and 10 µm (263.5±24.4 cells/well), but not at 0.1 µM (148.3±33.1 cells/well), compared to untreated controls (118.7±18.7 cells/well; Fig. 5.).

By contrast, the percentage of surviving RGC bearing neurites in cultures of axotomised retinal neurons did not significantly change after treatment with 0.1 µM (15.8±1.4%), 1 µM (10.0±1.9%) or 10 µM (10.9±1.9%) when compared to untreated controls (17.3±1.3%). The data suggest that VGF is a novel DPSC-derived neuroprotective, but not neuritogenic, factor for RGC at the optimal dose of 1 µm.

## Discussion

We have previously shown that rat DPSC promoted neurotrophin-mediated neuroprotection and neuritogenesis/axogenesis of axotomised RGC, both *in vitro* and *in vivo* [17]. Interestingly, rat DPSC promoted significantly greater neuroprotection and axon regeneration than rat BMSC, which reportedly protect RGC from death in models of optic nerve crush and glaucoma [14], [17]. We aimed to determine if the more potent neurotrophic properties were replicated by hDPSC, when compared to hBMSC and hAMSC and confirm that, like rat DPSC, hDPSC secreted multiple neuroprotective and axogenic NTF that protected RGC from death and promoted the growth of their neurites to a significantly greater extent than either hAMSC or hBMSC, which both have distinct NTF profiles as determined by RT-PCR and ELISA. Thus, we conclude that hDPSC may constitute an effective paracrine-mediated cellular therapy for retinal, and potentially other CNS injuries [17].

To determine the neuroprotective and neuritogenic effects of hDPSC-, hBMSC- and hAMSC- derived NTF, we cultured these human-derived stem cells with RGC. Similar to our previous results using rat stem cells [17], hDPSC and hBMSC stimulated RGC survival and neuritogenesis to levels greater than those seen in untreated control retinal cultures. Moreover, hDPSC were more neuroprotective and neuritogenic than hBMSC (although the former measure did not reach statistical significance), which corroborates our previous findings [17] as well as those of others showing greater functional restoration when hDPSC, as opposed to hBMSC, were transplanted into spinal cord lesion sites [32]. The neuroprotective and neuritogenic properties of hAMSC were significantly less than hDPSC and this was correlated with lower secreted levels of NGF, BDNF, NT-3, VEGF and GDNF observed by ELISA.

We used Fc-NTFR fusion protein blockers to examine the mechanism of the hDPSC-, hBMSC- and hAMSC-mediated neuroprotection and neuritogenesis. The neuroprotective effect of hDPSC was significantly reduced after the addition of each individual Fc-NTFR, confirming the contribution of stem cell-derived NGF, BDNF, NT-3, GDNF, VEGF and PDGF-AA/AB/BB. Similar observations were seen with hBMSC, suggesting that the mechanisms for neuroprotection/neuritogenesis are similar and that the reduced neuroprotective effect of hBMSC compared to hDPSC is explained by the reduced neurotrophic profile. Moreover, hAMSC had a similar RGC protective/regenerative potency to hBMSC yet, owing to a lack of BDNF secretion, Fc-TrKB had no effect on hAMSC-mediated RGC survival or neuritogenesis. Interestingly, Fc-PDGFBr reduced hDPSC−/hBMSC−/hAMSC-mediated neuroprotection despite no PDGF-AB/BB being detected in conditioned media by ELISA. This might be explained by the previous observation that PDGF-AB/BB was secreted at very low levels by hBMSC (40-fold <PDGF-AA) [15] and therefore expected to fall below the detectable range for the ELISA used. Thus, the efficacy of Fc-PDGFBr could be attributable either to the potency of low levels of PDGF-AB/BB or to PDGF-AB/BB secreted by glia present in the retinal cultures in response to hDPSC−/hBMSC−/hAMSC-derived growth factor stimulation.

The secretion of multiple NTF by hDPSC confirms previous findings showing NTF gene expression by hDPSC [19], [20], [32] as well as NTF secretion by rat-derived DPSC [17], [33] and neurotrophins by hDPSC [21]. In concert with our findings using rat DPSC [17], hDPSC secreted significantly more NGF and BDNF than hBMSC. Interestingly, compared to previous data on rat DPSC, hDPSC secreted higher titres of NGF but lower titres of BDNF [17]. Our results confirmed those in the literature on the extensive NTF secretory profile of hBMSC including NGF, BDNF, NT-3 and GDNF [17], [34]–[36], adding VEGF to the list of known secreted factors but failed to detect CNTF [32]. hAMSC secrete NGF, BDNF, NT-3, GDNF and VEGF [22], [23]. However in the present study, we have not detected the secretion of BDNF and NT-3, possibly because the titre was below the detectable range of the ELISA or that none was secreted in this study. Our results also support the findings of recent studies by others showing that hBMSC secrete PDGF-AA [15] and extend this observation to show that hDPSC and hAMSC share this property.

To further elucidate the relative neurotrophic activities of hDPSC, hBMSC and hAMSC, we conducted a RT-PCR array of 84 NTF/NTFR genes. The data showed that all three stem cell types have distinct NTF gene expression profiles. In particular, we found that hDPSC expressed prostaglandin E2 receptor (*ptger2*) at 6 and 10 fold higher than both hBMSC and hAMSC, respectively. Ptger2 stimulates the synthesis and release of neurotrophins from multiple cell types [37]–[39]. hDPSC also expressed over 100-fold lower interleukin-6 (*il6*) than hBMSC and hAMSC. The cytokine IL6 is neuroprotective after binding to the gp130 receptor [40] and promotes axon regeneration after activating the JAK/STAT3 pathway [41]. As a pro-inflammatory cytokine it is likely there are other IL6-mediated effects not fully realised in our *in vitro* paradigm. Finally, hBMSC express 6-fold higher fibroblast growth factor-9 (*fgf9*) than hDPSC, whereas hAMSC express over 700-fold higher *fgf9* than hDPSC. FGF9, unlike other FGF isoforms, stimulates the survival of RGC by binding to FGF receptor-3 [42], possibly highlighting a distinct mechanism for hAMSC-induced RGC neuroprotection. These data reinforce the notion that the stem cell origin is critical in determining their selection and application as cellular therapies for the treatment of particular neurological conditions.

NTF analyses by ELISA corresponded well with the RT-PCR microarray data, a point particularly well illustrated by the correlative BDNF and GDNF protein and mRNA data. Interestingly NGF values for protein and mRNA were paradoxical; demonstrating lower levels of NGF gene expression in hDPSC compared to hBMSC/hAMSC, while the titres of NGF protein in the conditioned medium from hDPSC cultures was significantly higher than that in the medium from hBMSC/hAMSC. These findings underline some discrepancies between gene expression and NTF protein secretion, which may be explained by differences in either the timing of sampling (PCR reflecting a snap-shot event while protein levels are cumulative) or an abundance of pre-existing stores of NGF in hDPSC.

The results presented here support the assertion that the RGC survival effects of the MSC are mediated in part by PDGF-AA [15]. Interestingly, inhibitors to the PDGFr did not significantly reduce RGC neuritogenesis, suggesting that MSC-derived PDGF is important for neuroprotection but not neuritogenesis in RGC.

RGC neurite outgrowth appeared to be particularly dependent on hDPSC-, hBMSC- and hAMSC-derived NGF. Thus, considering the enhanced secretion of NGF by hDPSC, may explain why hDPSC are more neuritogenic than hBMSC and hAMSC. BDNF and NT-3 are also neuritogenic for RGC, since neurite outgrowth was suppressed by the cognate Fc-TrK inhibitors. Noteworthy, hAMSC promoted very little neuritogenesis, although the response was significantly reduced when all three TrK receptors were simultaneously blocked.

Finally, this study is the first to identify VGF as a novel factor expressed at higher levels (>4 fold) in hDPSC than in hBMSC or hAMSC. VGF is a peptide present in the CNS and PNS [43] that protects motor neurons in animal models of amyotrophic lateral sclerosis [44] and increases the survival of cerebellar granule cells after serum deprivation [45]. We also showed that VGF was active in the retinal culture model at a concentration similar to that previously reported as active in other models of CNS injury [45]. In particular, VGF was significantly RGC neuroprotective, but not neuritogenic, suggesting that VGF may be involved in hDPSC-mediated RGC neuroprotection. Thus we propose that VGF may be a novel therapeutic NTF for RGC neuroprotection.

In conclusion, our results show that hDPSC is a more potent stem cell type for paracrine-mediated neuroprotection and regeneration of RGC than either hBMSC or hAMSC. The hDPSC-mediated neuroprotection and neuritogenesis is achieved through the paracrine effect of multiple secreted NTF, including PDGF (neuroprotection) and NGF (axon regeneration). Moreover, VGF is identified as a novel RGC neuroprotective factor expressed by hDPSC. hDPSC may represent an effective and advantageous cellular therapy for retinal nerve protection and repair.
